# Supplementary material for: GDF15 and ACE2 stratify COVID-19 patients according to severity while ACE2 mutations increase infection susceptibility
Source: Front Cell Infect Microbiol. 2022 Jul 22;12:942951. doi: 10.3389/fcimb.2022.942951 (PMC9355674; doi:10.3389/fcimb.2022.942951)
Supplement: Supplementary Table 1 — Primer sequences and annealing temperatures. [file Table_1.docx]

**Supplemental Table 1. Primer sequences and annealing temperatures.**

| **Gene** | **Forward primer (5’-3’)**  **Reverse primer (5’-3’)** | **Annealing temperature (˚C)** |
| --- | --- | --- |
| *GAPDH* | 5'-CCACTCCTCCACCTTTGACG-3'  5'-CTGGTGGTCCAGGGGTCTTA-3' | 60 |
| *18S* | 5'-GGACACGGACAGGATTGA CA-3'  5'-ACCCACGGAATCGAGAAAGA-3' | 60 |
| *ACE2* | 5'-TCCATTGGTCTTCTGTCACCCG-3'  5'-AGACCATCCACCTCCACTTCTC-3' | 60 |
| *HIF1A* | 5'-TCTTGGAAACGTGTAAAAGGATGC-3'  5'-CAGTCTACATGCTAAATCAGAGGGT-3' | 60 |
| *CDKN1A* | 5'-TGGAGACTCTCAGGGTCGAAA-3'  5'-GGCGTTTGGAGTGGTAGAAATC-3' | 61 |
| *CDKN2A* | 5'-TGAGCACTCACGCCCTAAGC-3'  5'-TAGCAGTGTGACTCAAGAGAAGCC-3' | 61 |
| *COX3* | 5’-ATGACCCACCAATCACATGC-3’  5’-ATCACATGGCTAGGCCGGAG-3’ | 55 |

**Supplemental Table 2. Primer sequences and annealing temperatures for assessment of mtDNA oxidation.**

| **Oxidized mtDNA** | **Forward primer (5’-3’)**  **Reverse primer (5’-3’)** | **Annealing temperature (˚C)** |
| --- | --- | --- |
| *Short fragment* | 5’-CAGCACAAAGTCCTGTGGAA-3’  5’-CGAAGAGGTAAGATCATCTGGT-3’ | 60 |
| *Long fragment* | 5’-CCTGAAACTTCAATGCCAAA-3’  5’-CGAAGAGGTAAGATCATCTGGT-3’ | 60 |
