# Supplementary material for: GDF15 and ACE2 stratify COVID-19 patients according to severity while ACE2 mutations increase infection susceptibility
Source: Front Cell Infect Microbiol. 2022 Jul 22;12:942951. doi: 10.3389/fcimb.2022.942951 (PMC9355674; doi:10.3389/fcimb.2022.942951)
Supplement: Supplementary Table 2 — Primer sequences and annealing temperatures for assessment of mtDNA oxidation. [file Table_2.docx]

| **Supplemental Table 3. Clinical and Biochemical Characteristics of non-ICU group** | | | |
| --- | --- | --- | --- |
|  | No-ICU group, n=21 | | |
| **Sociodemographic and history illness information** | | Mean | SD |
| Age (years) | | 71.3 | 7.28 |
| Sex (women), n (%) | | 9 | 42.9 |
| Smoking habit (Yes), n (%) | | 13 | 61.9 |
| Hypertension (Yes), n (%) | | 13 | 61.9 |
| T2DM (Yes), n (%) | | 10 | 47.6 |
| Obesity (Yes), n (%) | | 2 | 9.5 |
| COPD (Yes), n (%) | | 0 | 0 |
| Dyslipidaemia (Yes), n (%) | | 10 | 47.6 |
| **Biochemical parameters** | |  |  |
| GDF15 | | 1557 | 852 |
| ACE2 | | 6.43 | 1.75 |
| Leukocytes, 10^9^/L | | 6.99 | 2.01 |
| Neutrophil count, 10^9^/L | | 4.26 | 1.73 |
| Lymphocyte count, 10^9^/L | | 1.97 | 0.67 |
| Monocyte count, 10^9^ /L | | 0.57 | 0.19 |
| Eosinophils, , 10^9^ /L | | 0.16 | 0.09 |
| Haematids | | 4.45 | 0.55 |
| Hemoglobin, g/dL | | 13.7 | 1.45 |
| Hematocrit, % | | 40.7 | 4.56 |
| Mean Corpuscular Volume (MCV), f | | 91.8 | 4.60 |
| Mean Corpuscular Hemoglobin (MCH), pg | | 31.1 | 2.15 |
| Red Blood Cell Distribution Width (RDW), % | | 13.1 | 1.02 |
| Platelets, 10^9^/L | | 206 | 48.7 |
| Mean Platelet Volume (MPV), f | | 8.48 | 1.23 |
| Platelet Distribution width (PDW), % | | 16.1 | 0.66 |
| Neutrophils - Lymphocytes ratio | | 2.41 | 1.44 |
| Protombin ratio | | 1.27 | 0.96 |
| D-Dimer, ng/mL | | 172 | 177 |
| Glucose, mg/dL | | 124 | 64.7 |
| Urea, mg/dL | | 45.6 | 19.1 |
| Creatinine, mg/dL | | 1.02 | 0.48 |
| Aspartate aminotransferase (AST), U/L | | 19 | 4.31 |
| Alanine aminotransferase (ALT), U/L | | 17.3 | 8.16 |
| Gamma-glutamyl transferase (GGT), U/L | | 22.3 | 10.6 |
| Lactate dehydrogenase (LDH), U/L | | 204 | 31.3 |
| Ferritin, ng/mL | | 108 | 72.2 |
| C reactive protein (CRP), mg/L | | 0.63 | 0.97 |
| Triglycerides, mg/dL | | 132 | 49.8 |
| Nt_PRO BNP, pg/mL | | 385 | 868 |

Data shown is mean (SD), unless otherwise specified. The sample of the present analyses was n=21, corresponding to the non-ICU group. Abbreviations: SD: Standard desviation; ACE2: angiotensin 2-converting enzyme; GDF-15: growth differentiation factor 15; T2DM: Type 2 diabetes mellitus; COPD: chronic obstructive pulmonary disease; Hb: haemoglobin; VCM: mean corpuscular volume; PDW VPM: mean platelet volume; LDH: lactate dehydrogenase; GGT: gamma glutamyl transpeptidase; ALT: alanine aminotransferase; AST: aspartate aminotransferase; CPR: c-reactive protein; NT_PROBNP: brain natriuretic peptide.
