# Supplementary material for: GDF15 and ACE2 stratify COVID-19 patients according to severity while ACE2 mutations increase infection susceptibility
Source: Front Cell Infect Microbiol. 2022 Jul 22;12:942951. doi: 10.3389/fcimb.2022.942951 (PMC9355674; doi:10.3389/fcimb.2022.942951)
Supplement: Supplementary Table 3 — Clinical and Biochemical Characteristics of non-ICU group [file Table_3.docx]

|  | **Supplemental Table 4**. Correlation coefficient between GDF15, ACE2 and changes on biochemical parameters among non-ICU COVID19 patients. | | | | | | | | | | | | | | | | |  | |  |
| --- | --- | --- | --- | --- | --- | --- | --- | --- | --- | --- | --- | --- | --- | --- | --- | --- | --- | --- | --- | --- |
|  | | **GDF15** | | **ACE2** | | **Age** | **Ferritin** | **CRP** | | **ACE2^2–∆∆Ct^** | **HIF1A^2–∆∆Ct^** | **ACE2^2–∆∆Ct#^** | **HIF1A^2–∆∆Ct#^** | **P16^2–∆∆Ct#^** | **P21^2–∆∆Ct#^** | **mtDNA/**  **nDNA^#^** | **mtDNAox**  **^2–∆∆Ct#^** | | **TL**  **^2–∆∆Ct#^** | |
| **GDF15** | | 1.000 | | |  |  |  |  | |  |  |  |  |  |  |  |  | |  | |
| **ACE2** | | 0.304 | | | 1.000 |  |  | |  |  |  |  |  |  |  |  |  | |  | |
| **Age** | | **0.450*** | | | -0.026 | 1.000 |  | |  |  |  |  |  |  |  |  |  | |  | |
| **Ferritin** | | **0.480*** | | | 0.391 | -0.209 | 1.000 | |  |  |  |  |  |  |  |  |  | |  | |
| **CRP** | | 0.281 | | | 0.334 | -0.149 | **0.482*** | | 1.000 |  |  |  |  |  |  |  |  | |  | |
| **ACE2^2–∆∆Ct^** | | 0.395 | | | 0.370 | -0.281 | **0.862***** | | 0.330 | 1.000 |  |  |  |  |  |  |  | |  | |
| **HIF1A^2–∆∆Ct^** | | -0.163 | | | 0.021 | -0.330 | -0.128 | | -0.162 | -0.177 | 1.000 |  |  |  |  |  |  | |  | |
| **ACE2^2–∆∆Ct#^** | | 0.102 | | | 0.273 | -0.397 | 0.184 | | -0.052 | 0.157 | **0.704**** | 1.000 |  |  |  |  |  | |  | |
| **HIF1A^2–∆∆Ct#^** | | -0.262 | | | 0.104 | -0.041 | -0.161 | | -0.259 | **0.637**** | -0.257 | -0.062 | 1.000 |  |  |  |  | |  | |
| **P16 ^2–∆∆Ct#^** | | 0.118 | | | -0.657 | -0.473 | -0.532 | | 0.086 | 0.602 | 0.578 | 0.810 | -0.041 | 1.000 |  |  |  | |  | |
| **P21^2–∆∆Ct#^** | | -0.237 | | | -0.016 | -0.256 | -0.119 | | -0.134 | -0.124 | **0.656*** | **0.590*** | 0.087 | **0.983**** | 1.000 |  |  | |  | |
| **mtDNA/**  **nDNA** | | -0.178 | | | 0.056 | -0.121 | -0.187 | | **0.636**** | -0.196 | 0.109 | -0.218 | -0.091 | 0.225 | 0.024 | 1.000 |  | |  | |
| **mtDNAox**  **^2–∆∆Ct#^** | | **-0.495*** | | | 0.040 | -0.349 | -0.128 | | 0.196 | -0.138 | 0.287 | -0.050 | 0.184 | -0.238 | 0.326 | **0.576**** | 1.000 | |  | |
| **TL^2–∆∆Ct#^** | | | -0.271 | | 0.012 | -0.271 | -0.172 | | **0.488*** | -0.168 | 0.057 | 0.175 | -0.048 | 0.313 | 0.245 | **0.590**** | 0.317 | | 1.000 | |

Correlation coefficient and p-value, *p < 0.05; **p < 0.01; ***p < 0.001; 2^–∆∆Ct^ (delta delta CT, relative fold change expression in plasma); 2^–∆∆Ct^ # (delta delta CT, relative fold change expression in buffy coats); ACE2: angiotensin 2-converting enzyme; GDF15: growth differentiation factor 15; CRP: C reactive protein; HIF1A: hypoxia inducible factor 1 alpha; mtDNA/nDNA: ratio mitochondrial DNA to nuclear DNA; mtDNAox: mitochondrial DNA oxidation; TL: telomere length

|  | **Supplemental Table 4**. Correlation coefficient between GDF15, ACE2 and changes on biochemical parameters among non-ICU COVID19 patients. | | | | | | | | | | | | | | |  | | |  | |
| --- | --- | --- | --- | --- | --- | --- | --- | --- | --- | --- | --- | --- | --- | --- | --- | --- | --- | --- | --- | --- |
|  | | **GDF-15** | | **ACE2** | **ACE2^2–∆∆Ct^** | **ACE2**  **^2–∆∆Ct#^** | **HIF1A**  **^2–∆∆Ct^** | | **HIF1A**  **^2–∆∆Ct#^** | **Leukocytes** | **Neutrophil** | **Lymphocyte** | **Monocyte** | **Eosinophils** | **Basophils** | **Platelets** | **Neutro to Lymp ratio^1^** | | | |
| **GDF-15** | | | 1.000 |  |  |  | |  |  |  |  |  |  |  |  |  | |  | |  |
| **ACE2** | | | 0.304 | 1.000 |  |  | |  |  |  |  |  |  |  |  |  | |  | |  |
| **ACE2^2–∆∆Ct^** | | | 0.395 | 0.370 | 1.000 |  | |  |  |  |  |  |  |  |  |  | |  | |  |
| **ACE2^2–∆∆Ct#^** | | | 0.102 | 0.273 | 0.157 | 1.000 | |  |  |  |  |  |  |  |  |  | |  | |  |
| **HIF1A^2–∆∆Ct^** | | | -0.163 | 0.021 | -0.177 | **0.704**** | | 1.000 |  |  |  |  |  |  |  |  | |  | |  |
| **HIF1A^2–∆∆Ct#^** | | | -0.262 | 0.104 | **0.637*** | -0.062 | | -0.257 | 1.000 |  |  |  |  |  |  |  | |  | |  |
| **Leukocytes** | | | 0.163 | 0.396 | 0.363 | 0.189 | | -0.112 | 0.067 | 1.000 |  |  |  |  |  |  | |  | |  |
| **Neutrophil** | | | 0.242 | 0.394 | **0.478*** | 0.274 | | -0.071 | 0.068 | **0.929***** | 1.000 |  |  |  |  |  | |  | |  |
| **Lymphocyte** | | | -0.197 | 0.023 | -0.237 | -0.203 | | -0.155 | 0.103 | 0.378 | 0.022 | 1.000 |  |  |  |  | |  | |  |
| **Monocyte** | | | 0.204 | 0.329 | 0.201 | -0.019 | | -0.096 | -0.259 | **0.611**** | **0.528*** | 0.159 | 1.000 |  |  |  | |  | |  |
| **Eosinophils,** | | | 0.169 | 0.436 | -0.082 | **0.542*** | | 0.388 | 0.154 | 0.250 | 0.171 | 0.119 | 0.212 | 1.000 |  |  | |  | |  |
| **Basophils,** | | | -0.400 | -0.042 | -0.273 | -0.149 | | -0.151 | -0.204 | 0.337 | 0.220 | **0.433*** | -0.042 | -0.128 | 1.000 |  | |  | |  |
| **Platelets,** | | | **-0.557**** | -0.159 | -0.261 | 0.026 | | 0.124 | -0.426 | 0.289 | 0.079 | **0.576**** | 0.276 | -0.091 | **0.479*** | 1.000 | |  | |  |
| **Neutro to Lymp ratio^1^** | | | 0.413 | 0.302 | **0.629**** | 0.339 | | -0.028 | -0.077 | **0.500*** | **0.750***** | **-0.530*** | 0.341 | -0.004 | -0.078 | -0.329 | | 1.000 | |  |

Correlation coefficient and p-value, *p < 0.05; **p < 0.01; ***p < 0.001. _1_ neutrophils/ lymphocytes ratio; 2^–∆∆Ct^ (delta delta CT, relative fold change expression in plasma); 2^–∆∆Ct^ # (delta delta CT, relative fold change expression in buffy coats); ACE2: angiotensin 2-converting enzyme; GDF15: growth differentiation factor 15; HIF1A: hypoxia inducible factor 1 alpha; mtDNA/nDNA: ratio mitochondrial DNA to nuclear DNA; mtDNAox: mitochondrial DNA oxidation;

|  | **Supplemental Table 4**. Correlation coefficient between GDF15, ACE2 and changes on biochemical parameters among non-ICU COVID19 patients. | | | | | | | | | | | |  |  |
| --- | --- | --- | --- | --- | --- | --- | --- | --- | --- | --- | --- | --- | --- | --- |
|  | **GDF15** | **ACE2** | **ACE2**  **^2–∆∆Ct^** | **ACE2**  **^2–∆∆Ct#^** | **HIF1A**  **^2–∆∆Ct^** | **HIF1A**  **^2–∆∆Ct#^** | **mtDNAox**  **^2–∆∆Ct#^** | **mtDNA/**  **nDNA^#^** | **Glucose** | **Urea** | **Creatinine** | **TG** | **Nt_PRO**  **BNP** | **Prothrombin time** |
| **GDF15** | 1.000 |  |  |  |  |  |  |  |  |  |  |  |  |  |
| **ACE2** | 0.304 | 1.000 |  |  |  |  |  |  |  |  |  |  |  |  |
| **ACE2^2–∆∆Ct^** | 0.395 | 0.370 | 1.000 |  |  |  |  |  |  |  |  |  |  |  |
| **ACE2^2–∆∆Ct#^** | 0.102 | 0.273 | 0.157 | 1.000 |  |  |  |  |  |  |  |  |  |  |
| **HIF1A^2–∆∆Ct^** | -0.163 | 0.021 | -0.177 | **0.704**** | 1.000 |  |  |  |  |  |  |  |  |  |
| **HIF1A^2–∆∆Ct#^** | -0.262 | 0.104 | **0.637*** | -0.062 | -0.257 | 1.000 |  |  |  |  |  |  |  |  |
| **mtDNAox**  **^2–∆∆Ct#^** | -0.348 | -0.107 | -0.201 | -0.059 | -0.003 | -0.176 | 1.000 |  |  |  |  |  |  |  |
| **mtDNA/**  **nDNA^#^** | -0.178 | 0.056 | -0.196 | -0.218 | 0.109 | -0.091 | 0.149 | 1.000 |  |  |  |  |  |  |
| **Glucose** | **0.526*** | 0.398 | **0.839***** | 0.191 | -0.189 | **0.582*** | -0.279 | -0.318 | 1.000 |  |  |  |  |  |
| **Urea** | **0.727**** | 0.309 | **0.608**** | 0.095 | -0.155 | -0.110 | -0.411 | -0.245 | **0.633^**^** | 1.000 |  |  |  |  |
| **Creatinine** | **0.705**** | 0.310 | **0.736**** | 0.010 | -0.142 | 0.120 | -0.345 | 0.049 | **0.706**** | **0.813***** | 1.000 |  |  |  |
| **TG** | **0.486*** | 0.143 | 0.071 | -0.196 | -0.235 | -0.144 | 0.351 | -0.224 | 0.148 | 0.268 | 0.270 | 1.000 |  |  |
| **Nt_PRO**  **BNP** | **0.494*** | 0.351 | **0.889***** | 0.176 | -0.126 | -0.248 | -0.217 | -0.224 | **0.944***** | **0.665**** | **0.784***** | 0.161 | 1.000 |  |
| **Prothrombin time** | 0.172 | 0.024 | -0.177 | -0.086 | 0.075 | -0.131 | -0.256 | **0.628**** | -0.106 | -0.088 | 0.159 | -0.167 | -0.028 | 1.000 |

Correlation coefficient and p-value, *p < 0.05; **p < 0.01; ***p < 0.001. Abbreviations: TG; triglycerides; 2^–∆∆Ct^ (delta delta CT, relative fold change expression in plasma); 2^–∆∆Ct^ # (delta delta CT, relative fold change expression in buffy coats); ACE2: angiotensin 2-converting enzyme; GDF15: growth differentiation factor 15; HIF1A: hypoxia inducible factor 1 alpha; mtDNA/nDNA: ratio mitochondrial DNA to nuclear DNA; mtDNAox: mitochondrial DNA oxidation; NT_PROBNP: brain natriuretic peptide.

|  | **Supplemental Table 4**. Correlation coefficient between GDF15, ACE2 and changes on biochemical parameters among non-ICU COVID19 patients. | | | | | | | | | | | | | | | | |
| --- | --- | --- | --- | --- | --- | --- | --- | --- | --- | --- | --- | --- | --- | --- | --- | --- | --- |
|  | | **GDF15** | | **ACE2** | **ACE2**  **^2–∆∆Ct^** | **ACE2**  **^2–∆∆Ct#^** | **HIF1A**  **^2–∆∆Ct^** | **HIF1A**  **^2–∆∆Ct#^** | **AST** | **ALT** | **GGT** | **LDH** | **D-Dimer** | **HCT** | **Hb** | **RBC** | **Igg** |
| **GDF-15** | | | 1.000 |  |  |  |  |  |  |  |  |  |  |  |  |  |  |
| **ACE2** | | | 0.304 | 1.000 |  |  |  |  |  |  |  |  |  |  |  |  |  |
| **ACE2^2–∆∆Ct^** | | | 0.395 | 0.370 | 1.000 |  |  |  |  |  |  |  |  |  |  |  |  |
| **ACE2^2–∆∆Ct#^** | | | 0.102 | 0.273 | 0.157 | 1.000 |  |  |  |  |  |  |  |  |  |  |  |
| **HIF1A^2–∆∆Ct^** | | | -0.163 | 0.021 | -0.177 | **0.704**** | 1.000 |  |  |  |  |  |  |  |  |  |  |
| **HIF1A^2–∆∆Ct#^** | | | -0.262 | 0.104 | **0.637*** | -0.062 | -0.257 | 1.000 |  |  |  |  |  |  |  |  |  |
| **AST** | | | 0.056 | 0.110 | -0.183 | -0.079 | 0.138 | -0.127 | 1.000 |  |  |  |  |  |  |  |  |
| **ALT** | | | 0.291 | 0.005 | -0.080 | -0.123 | 0.040 | 0.021 | **0.537*** | 1.000 |  |  |  |  |  |  |  |
| **GGT** | | | 0.157 | 0.144 | -0.140 | -0.218 | -0.078 | -0.017 | 0.098 | **0.544*** | 1.000 |  |  |  |  |  |  |
| **LDH** | | | -0.213 | -0.187 | 0.186 | 0.165 | 0.198 | -0.305 | 0.007 | -0.155 | -0.079 | 1.000 |  |  |  |  |  |
| **D-Dimer** | | | **0.592*** | 0.422 | **0.748**** | 0.239 | -0.212 | -0.233 | -0.064 | 0.068 | 0.123 | 0.458 | 1.000 |  |  |  |  |
| **HCT** | | | **0.448*** | -0.425 | **-0.485*** | -0.373 | -0.181 | 0.037 | 0.284 | 0.028 | -0.191 | 0.184 | -0.249 | 1.000 |  |  |  |
| **Hb** | | | -0.402 | -0.219 | **-0.511*** | -0.308 | -0.033 | 0.103 | 0.223 | 0.014 | -0.199 | 0.090 | -0.343 | **0.912***** | 1.000 |  |  |
| **RBC** | | | -0.344 | -0.347 | -0.397 | -0.324 | -0.118 | 0.001 | 0.356 | -0.046 | -0.130 | 0.187 | -0.202 | **0.905***** | **0.779***** | 1.000 |  |
| **Igg** | | | -0.113 | -0.202 | -0.341 | -0.469 | -0.320 | -0.289 | 0.587 | 0.377 | -0.230 | -0.026 | -0.148 | 0.457 | 0.150 | 0.525 | 1.000 |

Correlation coefficient and p-value, *p < 0.05; **p < 0.01; ***p < 0.001. 2^–∆∆Ct^ (delta delta CT, relative fold change expression in plasma); 2^–∆∆Ct^ # (delta delta CT, relative fold change expression in buffy coats); ACE2: angiotensin 2-converting enzyme; GDF15: growth differentiation factor 15; HIF1A: hypoxia inducible factor 1 alpha; AST: aspartate aminotransferase; ALT: alanine aminotransferase; GGT: gamma glutamyl transpeptidase; LDH: lactate dehydrogenase; HCt: hematocrit; HB: hemoglobin; RBC: Red Blood Cells; Igg: Immunoglobulin G.
