# Supplementary material for: GDF15 and ACE2 stratify COVID-19 patients according to severity while ACE2 mutations increase infection susceptibility
Source: Front Cell Infect Microbiol. 2022 Jul 22;12:942951. doi: 10.3389/fcimb.2022.942951 (PMC9355674; doi:10.3389/fcimb.2022.942951)
Supplement: Supplementary Table 4 — Correlation coefficient between GDF15, ACE2 and changes on biochemical parameters among non-ICU COVID19 patients. [file Table_4.docx]

| **Supplemental Table 5. ACE2 genotypes frequency among the study population.** | | | | |
| --- | --- | --- | --- | --- |
|  | ICU  n=21  Mean (SD) | Non-ICU  n= 26  Mean (SD) | Control group  n=25  Mean (SD) | p-value |
| Genotype 0, n (%) | 4 (19.0) | 2 (9.10) | 9 (36.0) | **<0.001** |
| Genotype 1, n (%) | 17 (81.0) | 11 (50.0) | 3 (12.0) | **0.001** |
| Genotype 2, n (%) | 0 (0) | 7 (31.8) | 12 (48.0) | **<0.001** |
| Genotype 3, n (%) | 0 (0) | 2 (9.10) | 1 (4.0) |  |

Data shown is mean (SD), unless otherwise specified. The sample of the present analyses was n=72. Genotype 0 corresponds to subjects that did not carry any variant; Genotype 1 corresponds to subjects that carry at least one promoting variant; Genotype 2 corresponds to subjects that carry at least one protective variant; Genotype 3 corresponds to subjects that carry at least one promoting and one protective variant.

| **Supplemental Table 6. Association between ACE2 genotypes with circulating levels of GDF15 and ACE2** | | |
| --- | --- | --- |
|  | GDF15 | |
| **Exposures** | β (95% CI) | p-value |
| Genotype 0 | Ref. |  |
| Genotype 1 | -25.6 (-853; 802) | 0.951 |
| Genotype 2 | 196 (-629; 1021) | 0.636 |
| Genotype 3 | 282 (-1270; 1834) | 0.718 |
|  | ACE 2 | |
| **Exposures** | β (95% CI) | p-value |
| Genotype 0 | Ref. |  |
| Genotype 1 | -1.74 (-4.72; 1.23) | 0.245 |
| Genotype 2 | -1.68 (-4.71; 1.35) | 0.271 |
| Genotype 3 | -2.15 (-7.71; 3.42) | 0.443 |

Values shown are β (95% CI). Linear regression models were used to assess the association between ACE2 genotypes with GDF-15 and ACE2, adjusting for age, sex and group. Analyses were performed with n=72. Genotype 0 corresponds to subjects that did not carry any variant; Genotype 1 corresponds to subjects that carry at least one promoting variant; Genotype 2 corresponds to subjects that carry at least one protective variant; Genotype 3 corresponds to subjects that carry at least one promoting and one protective variant.
