# Supplementary material for: GDF15 and ACE2 stratify COVID-19 patients according to severity while ACE2 mutations increase infection susceptibility
Source: Front Cell Infect Microbiol. 2022 Jul 22;12:942951. doi: 10.3389/fcimb.2022.942951 (PMC9355674; doi:10.3389/fcimb.2022.942951)
Supplement: Supplementary Figure 1 — ACE2 polymorphisms exhibit similar fashion when expressed in A549 cells. A549 cells were transfected with either GFP-ACE2 WT, GFP-ACE2 polymorphisms or GFP alone. Then, ACE2 protein expression was analyzed by A) Western Blot with MA5-32307 antibody B) Immunocytochemistry (red) with either MA5-32307 antibody (left panel) or MAB933 antibody (right panel). Nuclei was stained with DAPI (blue). Transfected cells contain GFP (green). CT: secondary antibody control to detect unspecific binding. Images were acquired with Cell Observer-Zeiss. Scale bar: 50 μm [file Presentation_1.pptx]

## Slide 1
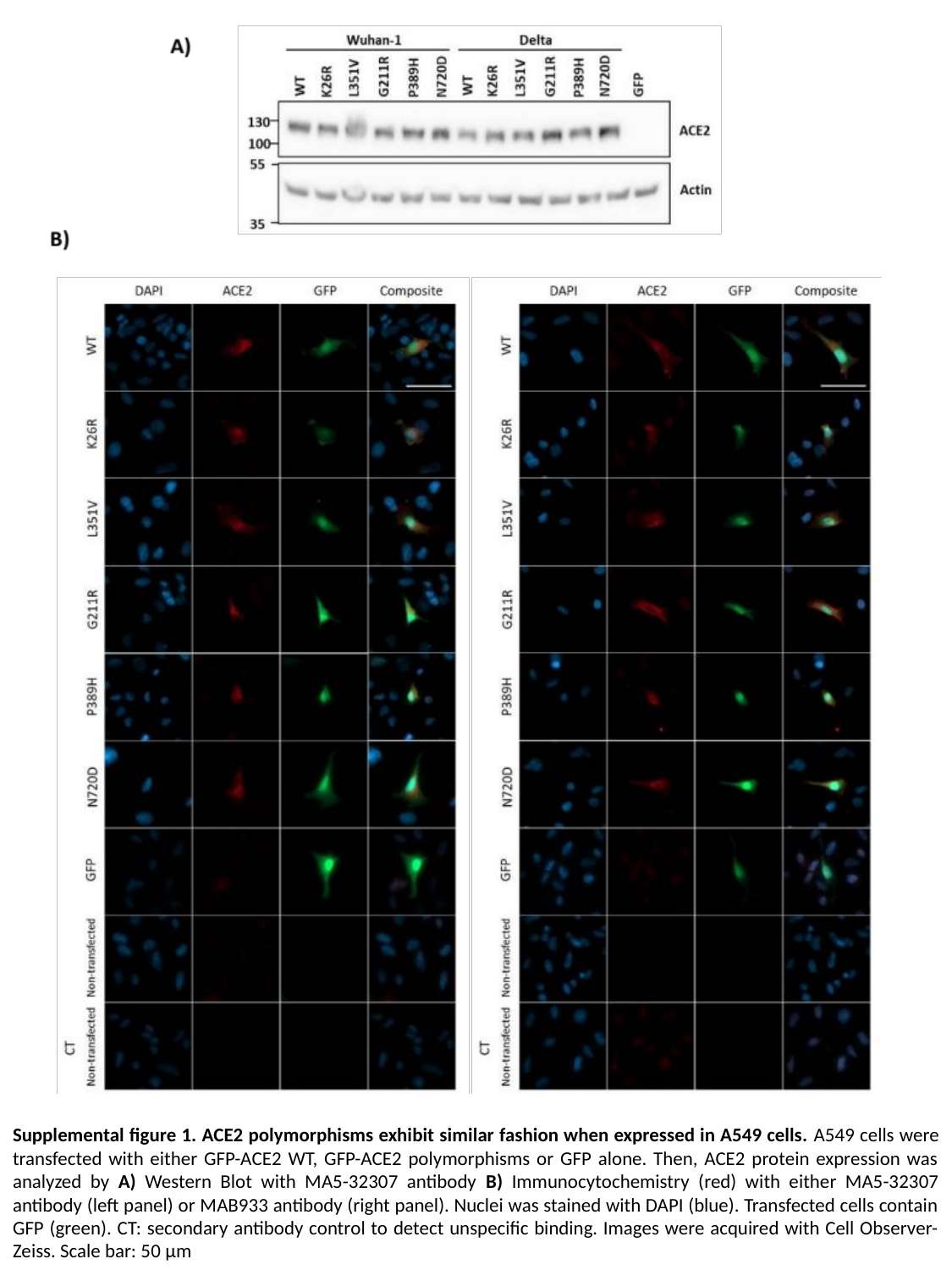

Supplemental figure 1. ACE2 polymorphisms exhibit similar fashion when expressed in A549 cells. A549 cells were transfected with either GFP-ACE2 WT, GFP-ACE2 polymorphisms or GFP alone. Then, ACE2 protein expression was analyzed by A) Western Blot with MA5-32307 antibody B) Immunocytochemistry (red) with either MA5-32307 antibody (left panel) or MAB933 antibody (right panel). Nuclei was stained with DAPI (blue). Transfected cells contain GFP (green). CT: secondary antibody control to detect unspecific binding. Images were acquired with Cell Observer-Zeiss. Scale bar: 50 μm

## Slide 2
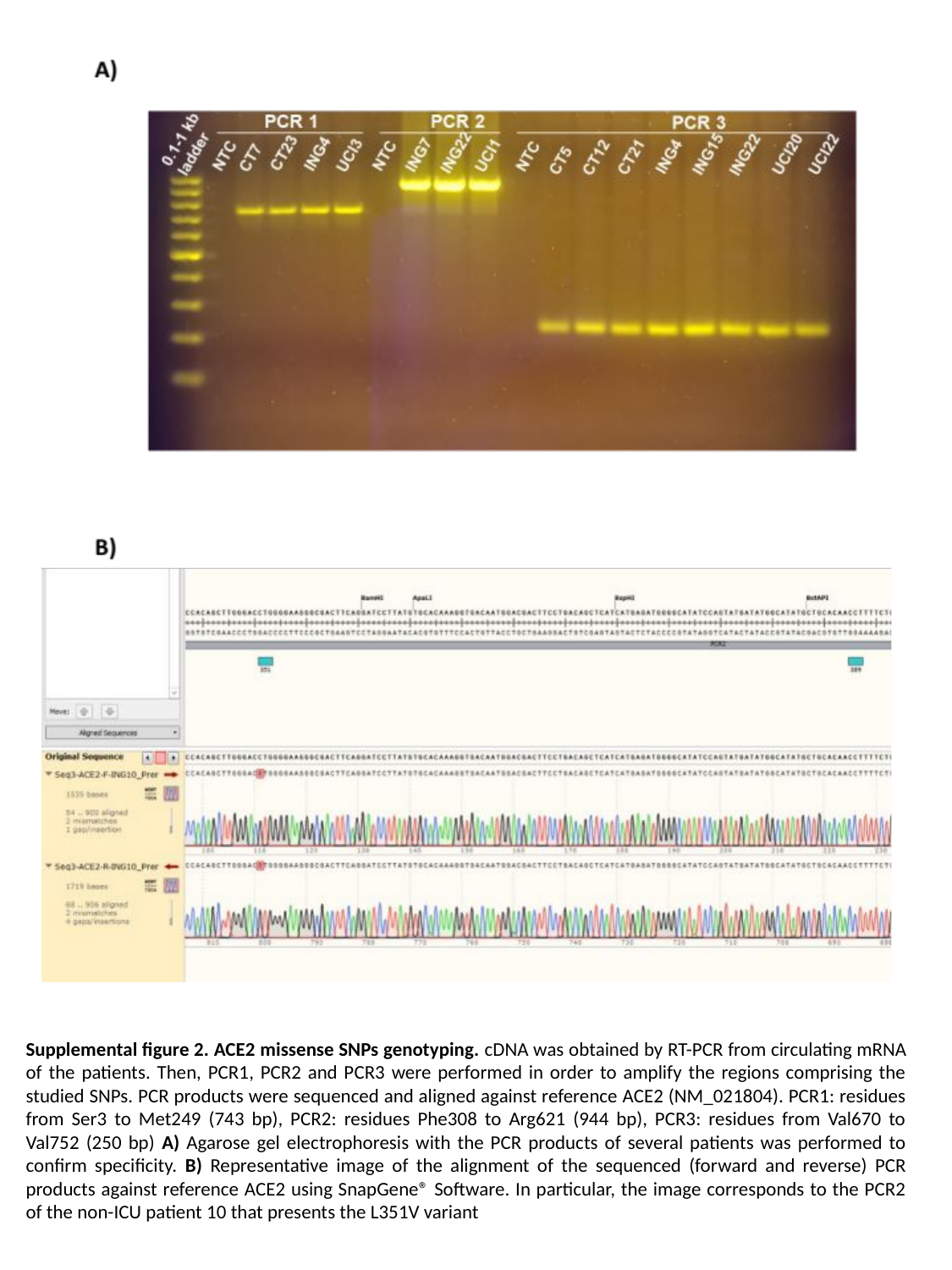

Supplemental figure 2. ACE2 missense SNPs genotyping. cDNA was obtained by RT-PCR from circulating mRNA of the patients. Then, PCR1, PCR2 and PCR3 were performed in order to amplify the regions comprising the studied SNPs. PCR products were sequenced and aligned against reference ACE2 (NM_021804). PCR1: residues from Ser3 to Met249 (743 bp), PCR2: residues Phe308 to Arg621 (944 bp), PCR3: residues from Val670 to Val752 (250 bp) A) Agarose gel electrophoresis with the PCR products of several patients was performed to confirm specificity. B) Representative image of the alignment of the sequenced (forward and reverse) PCR products against reference ACE2 using SnapGene® Software. In particular, the image corresponds to the PCR2 of the non-ICU patient 10 that presents the L351V variant

## Slide 3
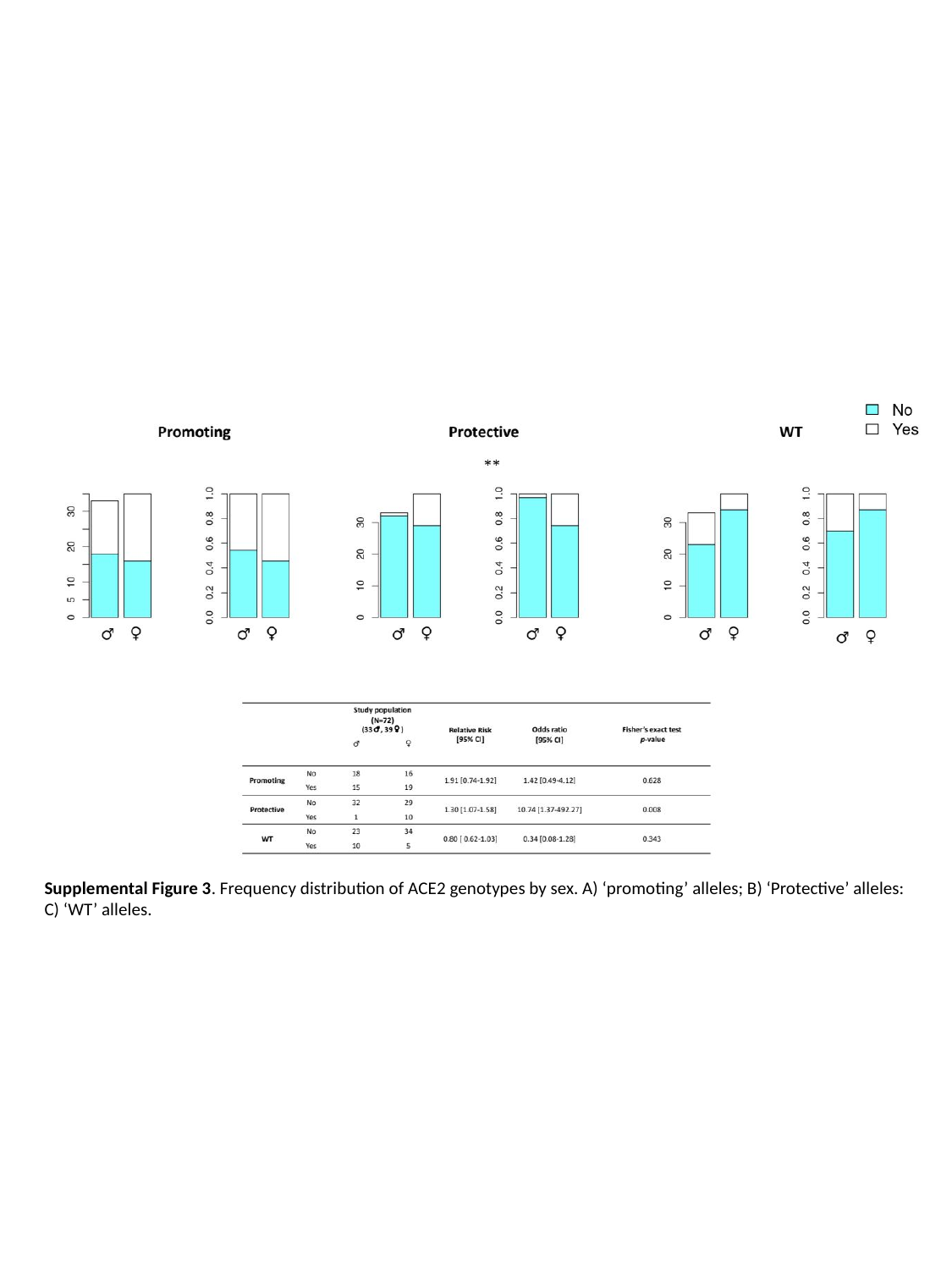

Supplemental Figure 3. Frequency distribution of ACE2 genotypes by sex. A) ‘promoting’ alleles; B) ‘Protective’ alleles: C) ‘WT’ alleles.

## Slide 4
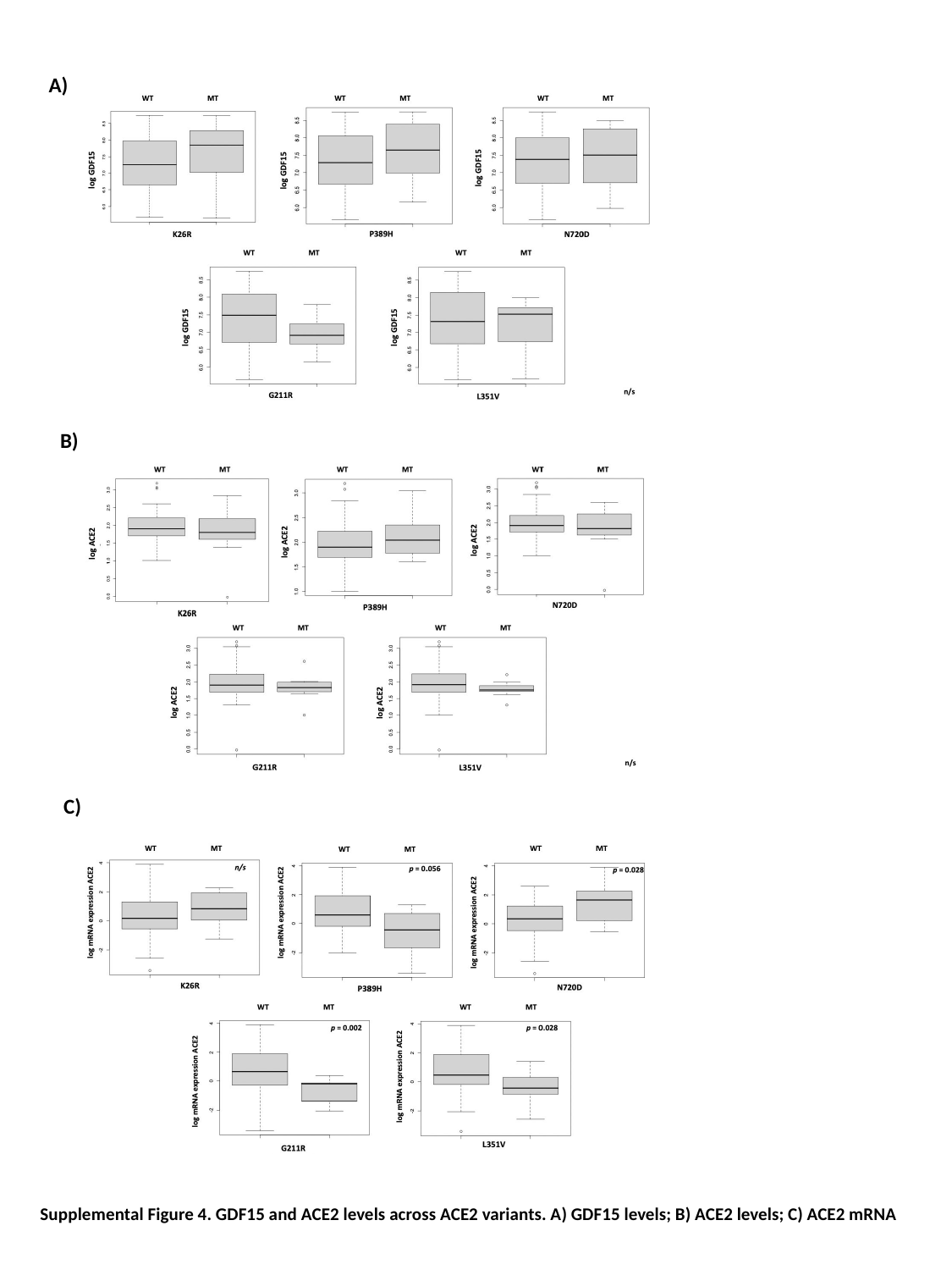

A)
B)
C)
Supplemental Figure 4. GDF15 and ACE2 levels across ACE2 variants. A) GDF15 levels; B) ACE2 levels; C) ACE2 mRNA
